# UNKAI: A Protein Functional Identity Prediction Model Based on ESM-C Latent Representations and the Attention Mechanism

**DOI:** 10.34133/csbj.0206

**Published:** 2026-08-14

**Authors:** Kotaro Ukai, Suguru Fujita, Tohru Terada

**Affiliations:** ^1^ Department of Biotechnology, Graduate School of Agricultural and Life Sciences, The University of Tokyo, Tokyo 113-8657, Japan.; ^2^ Department of Animal Resource Science, Graduate School of Agricultural and Life Sciences, The University of Tokyo, Tokyo 113-8657, Japan.

## Abstract

The rapid advancement of genome sequencing technologies has led to the accumulation of a vast number of protein sequences in public databases. However, a substantial proportion of these proteins remain functionally uncharacterized. Concurrently, the expansion of protein sequence data has enabled the development of protein language models (pLMs). By distilling billions of years of evolutionary history into a latent representational space, these models have acquired an unprecedented capacity to predict both the tertiary structures and functions of proteins. In this study, we developed a deep learning-based method to predict whether 2 proteins catalyze the same enzymatic reaction. Our approach leverages latent representations generated by ESM Cambrian (ESM-C), a state-of-the-art pLM, which are then processed through a neural network architecture integrating an attention mechanism. Our method outperformed existing approaches, including those based solely on full-length sequence similarity. Notably, it also surpassed our previous LightGBM-based model, which relied on structural similarity scores derived from AlphaFold-predicted models. Analysis of the attention weights reveals that our model autonomously highlights biologically significant sites, such as catalytic and binding residues. This demonstrates that integrating pLMs with attention mechanisms can enhance the accuracy and interpretability of protein function prediction while eliminating the need for manual feature engineering.

## Graphical Abstract

**Figure:**
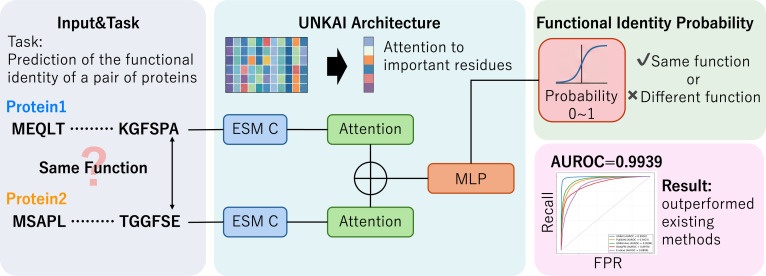

## Introduction

Owing to the rapid advancement of genome sequencing technologies, public databases—such as the Reference Sequence (RefSeq) collection of the National Center for Biotechnology Information (NCBI)—now contain hundreds of millions of protein sequences [1]. However, fewer than 0.2% of these proteins have been experimentally characterized [2]. To bridge this gap and facilitate the discovery of proteins with novel functions, efficient computational methods for protein function prediction are essential.

Traditionally, protein function prediction has relied on sequence alignment tools, such as BLAST, to identify homologous proteins under the premise that the sequence similarity implies functional similarity [3]. While many recent computational methods frame function prediction as a multi-class classification problem [4–7], we focus on a pairwise approach inspired by these alignment-based methods to determine whether 2 enzymes catalyze the same reaction involving the same substrate. We previously implemented this pairwise approach using a classical machine learning-based model referred to as “FUnctional identity of protein prediction by JoIning Sequence ANd structure feature (FUJISAN)” [8]. This model integrated full-length sequence similarity scores with structural similarity scores derived from the domain-level comparison of structural models predicted by AlphaFold2 [8,9]. FUJISAN demonstrated superior performance not only over a sequence identity-based baseline but also over models utilizing deep learning-based embeddings from DeepFRI [10] and ESM-2 [5]. Despite its high performance, FUJISAN’s reliance on structure-based features limits its scalability to massive datasets due to the substantial computational overhead associated with structure prediction, domain decomposition, and pocket detection. However, such limitations are inherent to a “hypothesis-driven feature engineering” approach, where specific biological features must be manually defined and computed.

Recent advances in protein language models (pLMs) and reaction-aware learning frameworks have substantially expanded the scope of computational enzyme function prediction [11–18]. In addition to conventional enzyme classification approaches based on EC numbers, several recent studies have focused on learning relationships between enzymes and their catalyzed reactions using large-scale biochemical databases and deep learning architectures. In particular, ReactZyme [18] introduced an enzyme–reaction retrieval framework based on Rhea and Swiss-Prot annotations, demonstrating that enzymatic function can be modeled directly at the reaction level rather than through predefined enzyme classes. Similar reaction-conditioned learning strategies and multimodal architectures integrating protein sequence representations, reaction fingerprints, and attention-based neural networks have further improved the prediction of enzyme–reaction associations and catalytic specificity. These studies highlight the growing importance of reaction-centric representations for understanding enzymatic function.

Despite these advances, most existing methods are designed to predict enzyme–reaction relationships or reaction assignments for individual proteins. In contrast, our objective is to determine whether 2 proteins share the same biochemical function, defined as catalyzing the same reaction on the same substrate. This pairwise formulation is closely aligned with practical homology-search workflows, where candidate proteins are first identified by sequence similarity and subsequently evaluated for functional equivalence. Therefore, our approach should be viewed as complementary to reaction retrieval frameworks, providing a scalable and accurate method for functional identity prediction among candidate enzyme pairs.

To overcome these limitations, we propose a novel deep learning framework that enables end-to-end prediction by integrating latent representations from ESM Cambrian (ESM-C) [19], a state-of-the-art pLM, with an attention pooling mechanism [20,21]. This mechanism enables the model to prioritize functionally critical residues during the prediction process. By employing a Siamese network architecture [22] with a learnable distance function, our method captures complex, nonlinear relationships between protein pairs without the need for explicit structural modeling or alignment. Our approach is intended to operate on candidate protein pairs identified through initial sequence similarity searches and is therefore designed to complement, rather than replace, conventional homology-based approaches. By leveraging contextual sequence representations and attention-based feature extraction, the model can capture functionally relevant signals that extend beyond simple sequence similarity. Unlike direct EC or Rhea classification models, our approach is designed as a pairwise functional inference framework. In practical applications, a query protein can be compared against a database of functionally annotated proteins, and candidate annotations can be ranked according to the predicted functional identity scores. This design is consistent with conventional homology-search workflows and allows flexible incorporation of newly annotated proteins without retraining the model. This study demonstrates that our end-to-end approach not only streamlines the computational pipeline but also achieves superior accuracy in predicting protein functional identity.

## Materials and Methods

### Dataset

In this study, protein function is defined by the Rhea ID, which is uniquely associated with a specific pair of substrate and product [23]. Consequently, a protein pair with identical function possesses the same Rhea ID, indicating that both proteins catalyze the same reaction involving the same chemical transformation. Following the methodology of our previous study [8], we retrieved 232,692 sequences annotated with Rhea IDs from the Swiss-Prot database [2]. An all-against-all BLAST search was performed on this sequence set, and all hits with *E* values less than 10 were retrieved. From these, only pairs consisting of proteins with up to 2,048 residues were retained for further analysis to accommodate the sequence length constraint of ESM-C. Subsequently, these protein pairs were categorized into 2 groups: positive pairs, consisting of proteins sharing the same Rhea ID, and negative pairs, consisting of proteins with distinct Rhea IDs. From each group, 100,000 pairs were randomly sampled to construct the final dataset.

A protein pair was regarded as functionally identical if the 2 proteins shared at least one Rhea identifier. Protein pairs without any shared Rhea identifiers were considered functionally different.

### Model architecture

**Fig. 1.**
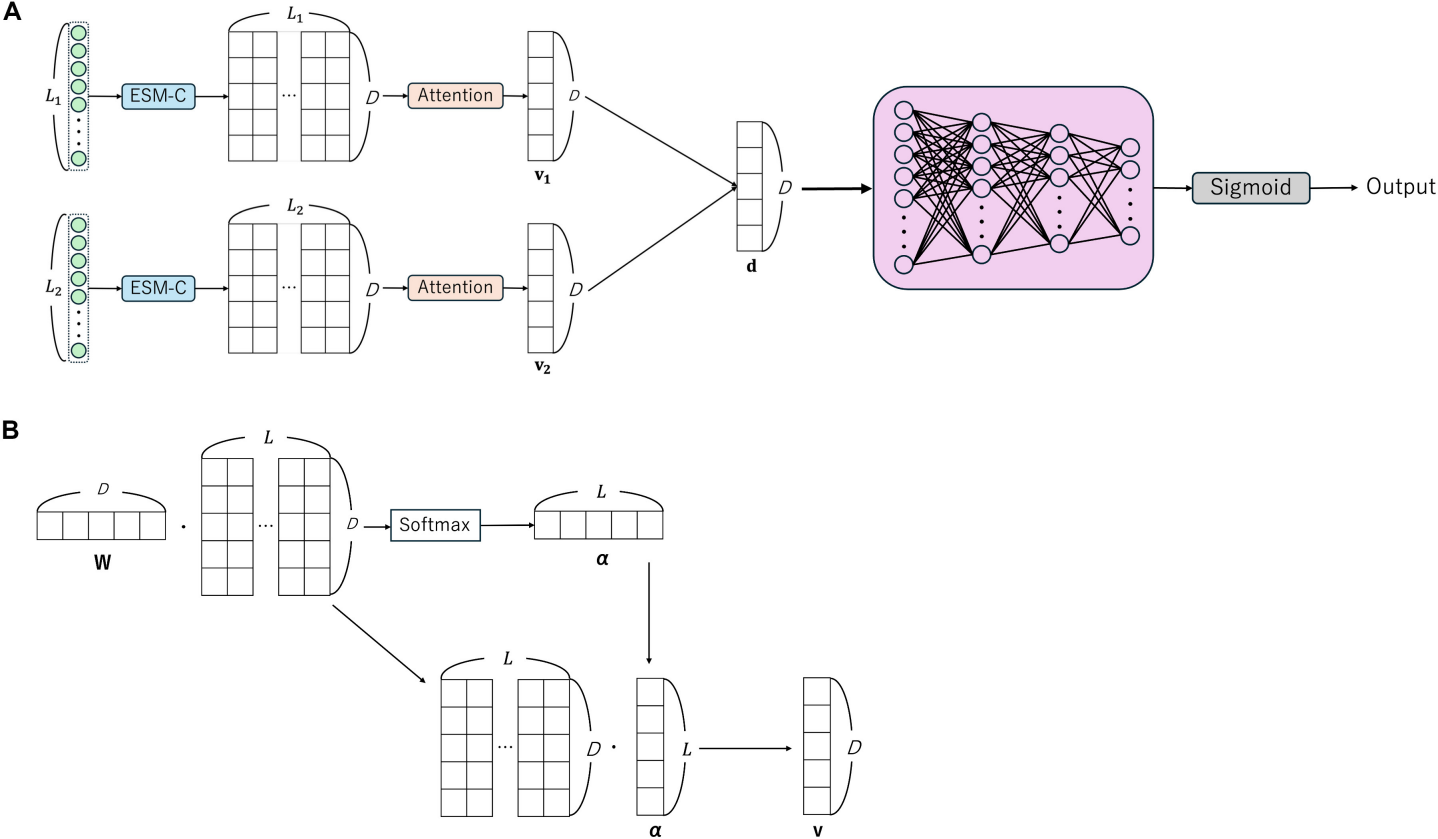
Model architecture. (A) Overall architecture of the proposed model, UNKAI, comprising an embedding layer, an attention pooling layer, and an MLP classifier. In the embedding layer, amino acid sequences of the input protein pair (proteins 1 and 2) are converted to ESM-C latent representations with dimensions of $D\times L_{1}$ and $D\times L_{2}$, where D=2,560 and $L_{1}$ and $L_{2}$ denote the sequence lengths. These representations are aggregated into fixed-size vectors $v_{1}$ and $v_{2}$ within the attention layer. The element-wise absolute difference d (where $d_{k}=\left|v_{1,k}-v_{2,k}\right|$) is then calculated and fed into the MLP classifier. The final MLP output is passed through a sigmoid function to obtain the probability of functional identity. (B) Schematic representation of the computation within the attention pooling layer.

Adopting a Siamese network architecture [22], the proposed model comprises 3 main components: an embedding layer, an attention pooling layer, and a multi-layer perceptron (MLP) classifier (Fig. 1A). In the embedding layer, the pretrained pLM, ESM-C [19], is employed to generate a latent representation of the input sequence with dimensions of D×L. Here, D and L denote the fixed dimensionality of the latent representation (D=2,560) and the sequence length, respectively. Subsequently, the variable-length latent representation $X\in {\mathbb{R}}^{D\times L}$ is aggregated into a fixed-size vector $v\in {\mathbb{R}}^{D}$ within the attention pooling layer, as illustrated in Fig. 1B. Let $x_{j}\in {\mathbb{R}}^{D}$ be the jth column vector of X corresponding to the embedding of residue j. For each $x_{j}$, an attention logit $\;e_{j}$ is computed via a linear transformation:

$$e_{j}=w_{\text{attn}}^{T}x_{j}+b_{\text{attn}}$$ (1)

Here, $w_{\text{attn}}\in {\mathbb{R}}^{D}$ and $b_{\text{attn}}$ are learnable parameters. The attention weights $\alpha_{j}$ are obtained by normalizing the logits $e_{j}$ using the softmax function:

$$\alpha_{j}=\frac{\text{exp}\left(e_{j}\right)}{\sum_{k=1}^{L}\text{exp}\left(e_{k}\right)}$$ (2)

Finally, the aggregated feature vector v is computed as the weighted sum of the residue embeddings:

$$v=\sum_{j=1}^{L}\alpha_{j}x_{j}$$ (3)

This process is applied independently to each protein of the input pair using shared parameters, yielding 2 representative vectors $v_{1}$ and $v_{2}$, corresponding to input 1 and input 2, respectively. To capture the relationship between the 2 proteins, the element-wise absolute difference $d\in {\mathbb{R}}^{D}$ between $v_{1}$ and $v_{2}$ is calculated and fed into the MLP classifier:

$$d_{k}=\left|v_{1,k}-v_{2,k}\right|,\;k=1,\cdots ,D$$ (4)

where $v_{1,k}$ and $v_{2,k}$ denote the kth elements of $v_{1}$ and $v_{2}$, respectively. The MLP classifier consists of 4 fully connected layers. The input dimension is 2,560, matching the dimensionality of d, followed by 3 hidden layers with 1,599, 781, and 117 units, respectively. Each layer is followed by batch normalization, a ReLU activation, and a dropout layer with a rate of 0.302766. The final MLP output is passed through a sigmoid function to obtain the probability of functional identity, constrained between 0 and 1. We used the ESM-C 6B model to generate residue-level embeddings. The ESM-C parameters were not fine-tuned and were kept fixed during training.

### Model training and hyperparameter optimization

The positive (identical function) and negative (distinct function) pair datasets were independently split into training (70%), validation (15%), and test (15%) subsets. Subsequently, the corresponding subsets from both groups were combined and randomly shuffled to form the final training, validation, and test sets. This procedure ensured that each set contained an equal number of positive and negative pairs, providing a balanced distribution for both model training and evaluation. The model was trained using the Adam optimizer [24] with a learning rate of $3.33\times 10^{-4}$ and the binary cross-entropy (BCE) loss function, defined as follows:

$$L_{\mathrm{BCE}}=-\frac{1}{N}\sum_{i=1}^{N}\left\{y_{i}\;\text{log}\left({\hat{y}}_{i}\right)+\left(1-y_{i}\right)\text{log}\left(1-{\hat{y}}_{i}\right)\right\}$$ (5)

where N denotes the number of samples in a batch, $y_{i}\in \left\{0,1\right\}$ is the ground-truth label, and ${\hat{y}}_{i}$ represents the predicted probability of functional identity for the ith pair. Training was performed for 10 epochs with a batch size of 64. Within each batch, sequences were padded to a uniform length, and an attention mask was applied to ensure that padded positions did not contribute to the attention weights or the resulting aggregated representations. The model achieving the highest F1 score (see below) on the validation set was saved.

To maximize the model’s predictive performance, hyperparameter optimization was performed via Bayesian optimization using the Optuna framework [25]. The search space encompassed the number of units in the 3 hidden layers ($n_{1},n_{2},n_{3}$), the dropout rate, and the learning rate. We explored the following ranges for hyperparameter optimization: hidden layer sizes $n_{1}\in \left[512,2048\right],n_{2}\in \left[256,n_{1}\right],$ and $n_{3}\in \left[64,n_{2}\right]$; dropout rate between 0.1 and 0.5; and learning rate from $10^{-5}$ to $10^{-2}$, the latter of which was sampled log-uniformly. A total of 50 optimization trials were conducted, with the objective of maximizing the F1 score on the validation set. To improve computational efficiency, we employed a Median pruner strategy to early-terminate unpromising trials. The final model was trained using the optimal hyperparameter configuration identified during this process and subsequently evaluated on the independent test set. The resulting model is named UNKAI (protein fUNKtionAl Identity prediction). In Japanese, *unkai* refers to a “sea of clouds”, a breathtaking phenomenon often viewed from the summit of Mt. Fuji (*Fujisan*). This name symbolizes our goal: Just as a sea of clouds extends far beyond the mountain peak, UNKAI is designed to be applicable to a more diverse range of proteins than our previous model, FUJISAN. All experiments were performed using a workstation equipped with an AMD EPYC 9354/3.25GHz + RTX 5080 (128 GB memory). Model training required approximately 4 d to complete under the described settings.

### Performance evaluation

We used several standard metrics to assess the model’s performance: accuracy (ACC), precision (PRE), recall (REC), false positive rate (FPR), and F1 score (F1). These metrics are defined based on the confusion matrix elements as follows:

$$\mathrm{ACC}=\frac{\mathrm{TP}+\mathrm{TN}}{\mathrm{TP}+\mathrm{FP}+\mathrm{TN}+\mathrm{FN}}$$ (6)

$$\mathrm{PRE}=\frac{\mathrm{TP}}{\mathrm{TP}+\mathrm{FP}}$$ (7)

$$\mathrm{REC}=\frac{TP}{\mathrm{TP}+\mathrm{FN}}$$ (8)

$$\mathrm{FPR}=\frac{\mathrm{FP}}{\mathrm{FP}+\mathrm{TN}}$$ (9)

$$F1=\frac{2\times \mathrm{PRE}\times \mathrm{REC}}{\mathrm{PRE}+\mathrm{REC}}$$ (10)

where TP, FP, TN, and FN represent the numbers of true positives, false positives, true negatives, and false negatives, respectively. Additionally, we utilized the area under the receiver operating characteristic curve (AUROC) and the area under the precision–recall curve (AUPR) to benchmark our model against other existing methods.

### Evaluation of the correlation between attention weights and functional annotations

To investigate whether the attention mechanism captures the functional residues of a protein, we extracted experimentally validated functional annotations—including “Active site”, “Binding site”, “Modified residue”, “Natural variant”, and “Site”—for the 59,989 proteins in the test set that carry at least one such annotation from the UniProt database [2]. Let $F_{A}$ denote the set of residue indices associated with a specific functional annotation A within a protein sequence. To perform the residue-level binary classification analysis, we assigned a binary label $y_{A,j}$ to each residue j as follows:

$$y_{A,j}=\left\{\begin{array}{c} 1\;\left(j\in F_{A}\right)\\ 0\;\left(j\notin F_{A}\;\right) \end{array}\right.$$ (11)

By treating attention weights $\alpha_{j}$ as classification scores, residue j is classified as functional for annotation A when $\alpha_{j}$ is above a threshold. The efficacy of this approach was independently quantified for each annotation A via ROC analysis to assess the discriminative power of the attention mechanism across different functional categories.

Since the attention weight profiles exhibited distinct peaks along the protein sequences (as shown later in the Results and Discussion section), we analyzed the positional proximity between these peaks and the known functional residues. Let $I_{\mathrm{top}}$ denote the set of residue indices with the highest 1% of attention weights within each protein sequence. As a baseline for comparison, we generated a control set $I_{\mathrm{rand}}$ by randomly sampling indices from the entire sequence without replacement, ensuring that it contained the same number of residues as $I_{\mathrm{top}}$. The minimum sequence distance from residue $j\in I_{X}$ ($X\in \left\{\mathrm{top},\text{rand}\right\}$) to the functional residues in $F_{A}$ was defined as follows [26]:

$$d_{X}\left(j,F_{A}\right)=\underset{j^{\prime }\in F_{A}}{\text{min}}\left|j-j^{\prime }\right|$$ (12)

We then calculated the probability mass function $P_{X,A}\left(k\right)$, representing the probability that $d_{X}\left(j,\;F_{A}\right)=k$ across all protein sequences in the test set.

### Evaluation of the correlation between attention weights and evolutionary conservation

We also examined the correlations between the attention weights and evolutionary conservation for the proteins in the test set. The evolutionary conservation of residue j was quantified using Shannon entropy $H\left(j\right)$, calculated from a multiple sequence alignment (MSA) generated by MMseqs2 [27] as follows:

Hj=−∑a∈Apa,jlog2pa,j (13)

where A denotes the set of the 20 standard amino acids, and $p_{a,j}$ represents the frequency of occurrence of amino acid a in the column corresponding to residue j. Insertions (represented as lowercase in the A3M format) and gaps (“-“ and “.”) were excluded from the entropy calculation. The correlation between the attention weights and the entropies was evaluated via Spearman’s rank correlation coefficient ρ [28], which is defined as:

ρ=1−6∑j=1Ldj2LL2−1 (14)

where L is the sequence length, and $d_{j}$ denotes the difference between the ranks of the attention weight and the entropy for residue j.

### Evaluation of the predictive performance of UNKAI on unseen EC subclasses

Leave-one-EC-subclass-out evaluation was performed to assess the ability of UNKAI to generalize to unseen functional categories. Here, EC subclasses refer to the third level of the Enzyme Commission (EC) classification system (e.g., EC 2.7.1 and EC 3.6.4). Rhea-to-EC mappings were obtained from the official Rhea database. Five EC subclasses were selected for evaluation because they were well represented across the original training, validation, and test datasets, thereby providing sufficient numbers of protein pairs for model training and assessment. For each evaluation, all protein pairs associated with the selected EC subclass were removed from both the training and validation datasets and used exclusively for testing. Proteins annotated with multiple Rhea identifiers were assigned to EC subclasses according to the corresponding Rhea-to-EC mappings.

### Evaluation on sequence identity-based splits

One concern regarding the original random pair-level split is that proteins with high sequence similarity may be distributed across the training, validation, and test datasets, potentially resulting in optimistic performance estimates. To evaluate the generalization ability of UNKAI under more stringent conditions, we constructed additional sequence identity-based datasets. First, all proteins were clustered according to sequence similarity using MMseqs2 at a 40% sequence identity threshold, and the resulting clusters were assigned to the training, validation, and test datasets before generating protein pairs. Positive and negative protein pairs were then independently generated, and 50,000 pairs were randomly sampled for each class while satisfying the following constraints. Reverse duplicate pairs (A–B/B–A) were excluded because both yield identical input features (|v₁−v₂|). Furthermore, to prevent the model from memorizing specific proteins or families, strict sampling constraints were imposed independently within each dataset split (training, validation, and test sets): (a) Any individual protein accession appeared at most 10 times; (b) pairs involving proteins from a given sequence cluster were capped at 80; (c) identical cluster pairs were limited to a maximum of 15 occurrences; and (d) intra-cluster pairs accounted for no more than 10% of the pairs within that split. Positive and negative protein pairs were strictly balanced across all datasets. Two distinct evaluation scenarios were established. In the strict setting, protein pairs were constructed exclusively using proteins from clusters assigned to the corresponding dataset split, thereby evaluating the model’s ability to generalize to completely unseen sequence clusters. In the seen–unseen setting, each test pair consisted of one protein from a training cluster and one protein from an unseen cluster, representing a more practical scenario where only one protein is novel. The prediction performance was evaluated independently on both test datasets using the classification threshold that maximized the F1 score on the respective validation datasets.

## Results and Discussion

### Predictive performance of UNKAI

In this study, we developed a deep learning-based model, UNKAI, designed to predict the functional identity of protein pairs—specifically, whether the 2 proteins catalyze the same enzymatic reaction involving the same substrate. To construct the dataset, an all-against-all BLAST search was performed on Swiss-Prot sequences associated with Rhea IDs, which provide unique identifiers for enzymatic reactions. From the pairs exhibiting detectable sequence similarity (*E* value < 10), we randomly selected 100,000 pairs with the same Rhea ID and 100,000 pairs with different Rhea IDs. The resulting dataset was partitioned into the training, validation, and test subsets at a ratio of 70:15:15. The model was trained for 10 epochs, during which stable convergence was observed (Fig. S1). We then assessed its predictive performance and benchmarked it against our previous model, FUJISAN, and other existing methods.

**Fig. 2.**
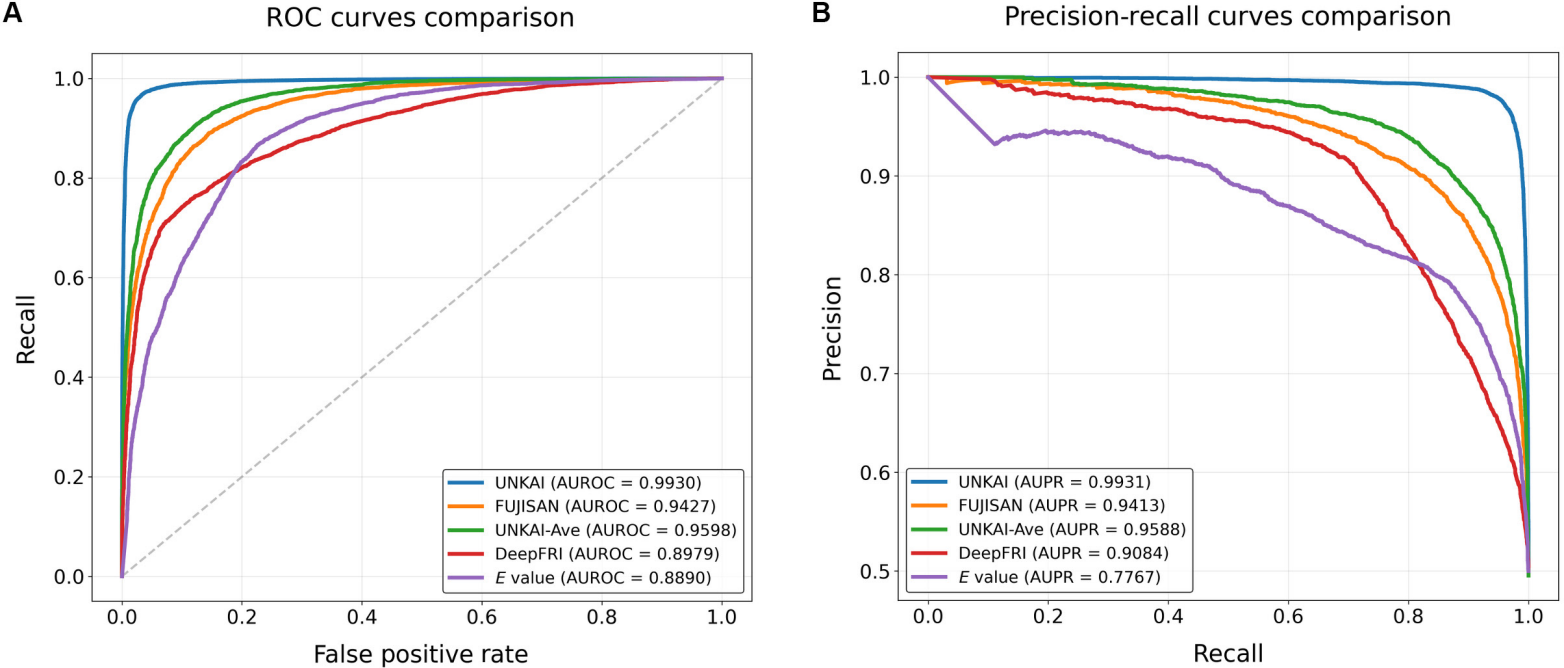
Comparison of predictive performance. (A) Receiver operating characteristic (ROC) curves and (B) precision–recall (PR) curves for UNKAI and benchmark methods. AUROC and AUPR values for each method are provided in parentheses within the legend. The dashed diagonal line in (A) represents the performance of a random classifier.

Figure 2 presents a comparison of the ROC and PR curves for these models. Detailed performance metrics, including AUROC and AUPR values, are summarized in Table 1. The results for FUJISAN, Deep-FRI, and the *E* value-based baseline were obtained from our previous study [8]. To compare the prediction performance of DeepFRI with our models, we calculated the cosine similarity between the output vectors for protein pairs, as described in [29]. The ROC and PR curves of UNKAI consistently surpassed those of existing methods, including FUJISAN, achieving AUROC and AUPR values of 0.9939 and 0.9931, respectively. Furthermore, UNKAI exhibited the best performance across all evaluation metrics among the compared methods. These results clearly demonstrate that UNKAI significantly outperformed the existing models in functional identity prediction.

**Table 1.** Predictive performance of UNKAI on the original random pair-level split and comparison with existing methods

Method	ACC	PRE	REC	FPR	F1	AUROC	AUPR
UNKAI	0.9966	0.9687	0.9643	0.0311	0.9665	0.9930	0.9931
UNKAI-Ave	0.8916	0.8651	0.9279	0.1447	0.8954	0.9598	0.9588
FUJISAN ^a^	0.8705	0.8724	0.8705	0.1335	0.8728	0.9427	0.9413
DeepFRI ^a^	0.8088	0.7977	0.8316	0.2145	0.8143	0.9067	0.9067
*E* value ^a^	0.8191	0.7858	0.8775	0.2392	0.8291	0.8890	0.7767

^a^ Cited from Ref. [8].

To evaluate the efficacy of the attention mechanism incorporated into UNKAI, we constructed a variant model, designated as UNKAI-Ave, in which average pooling was employed instead of attention pooling. As shown in Fig. 2 and Table 1, the performance of UNKAI-Ave was significantly degraded by the replacement of the attention mechanism, yielding AUROC and AUPR values of 0.9600 and 0.9590, respectively. These scores slightly exceed those of FUJISAN (AUROC: 0.9427, AUPR: 0.9413), confirming that using ESM-C latent representations as input for an MLP-based binary classifier is sufficient to achieve high baseline performance. However, these results highlight that the attention mechanism makes a significant contribution to further enhancing prediction accuracy. In the next section, we investigate how the attention mechanism captures functionally important regions within a protein sequence.

### Correlation between attention weights and functional annotations

**Fig. 3.**
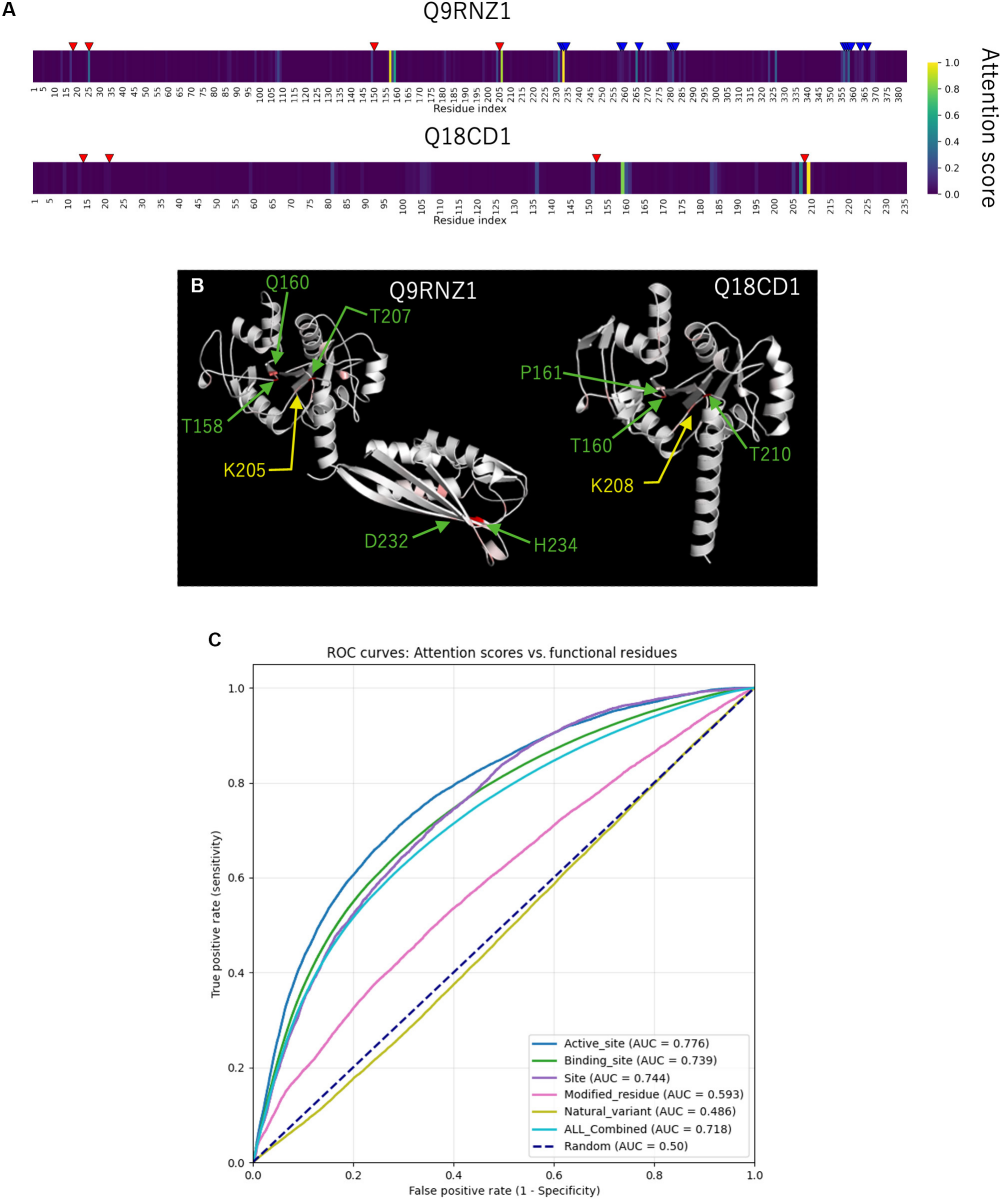
(A) Attention weight heatmaps for proteins Q9RNZ1 and Q18CD1. Functional annotations are indicated by red (site) and blue (binding site) triangles above the heatmaps. (B) AlphaFold-predicted structures of Q9RNZ1 and Q18CD1, with residues colored on a gradient from white (low) to red (high) based on their attention weights. Key residues with high attention weights are labeled and indicated with arrows. (C) Receiver operating characteristic (ROC) curves for the prediction of specific functional annotations based on attention weights. AUROC values are provided in parentheses within the legend. The dashed diagonal line represents the performance of a random classifier.

In the attention layer of UNKAI, a weighted sum of residue embeddings over the sequence length is computed to obtain a fixed-length aggregated feature vector [see Eq. (3)]. This architecture suggests that residues critical for functional identity are likely to be assigned larger attention weights. For example, UNKAI correctly predicted the functional identity of the protein pair Q9RNZ1 and Q18CD1, which share the same Rhea ID (13429). Notably, residues T158, Q160, K205, T207, D232, and H234 of Q9RNZ1, as well as T160, P161, K208, and T210 of Q18CD1, exhibited high attention weights (Fig. 3A). In the AlphaFold-predicted structures, the highlighted residues of Q18CD1 (T160, P161, K208, and T210) and a subset of those in Q9RNZ1 (T158, Q160, K205, and T207) are spatially situated within the corresponding cavities of each protein (Fig. 3B). Among these, K205 (Q9RNZ1) and K208 (Q18CD1) are specifically annotated as “Site” in UniProt, with the functional description: “position for the nucleophilic attack of MEP”. Interestingly, D232 and H234 of Q9RNZ1, which also exhibit high attention weights, are located in a separate domain. They are annotated as “Binding sites” for a different reaction (Rhea ID: 23864), suggesting that this additional domain catalyzes a reaction specific to Q9RNZ1. Based on these observations, we systematically assessed whether known functional sites could be predicted using the attention weights assigned to the proteins in the test set.

Figure 3C presents the ROC curves for the prediction of functional annotations: “Active site”, “Binding site”, “Modified residue”, “Natural variant”, and “Site”. The attention weights effectively captured “Active site”, “Binding site”, and “Site”, achieving AUROC values of 0.7760, 0.7395, and 0.7444, respectively. In contrast, the predictive performance was notably lower for “Modified residue” (AUROC: 0.5930) and “Natural variant” (AUROC: 0.4858). These results indicate that while the attention weights capture functional sites reasonably well, they do so with varying degrees of efficacy across categories. This further suggests that “Modified residue” and “Natural variant” may be less critical for the determination of functional identity.

**Fig. 4.**
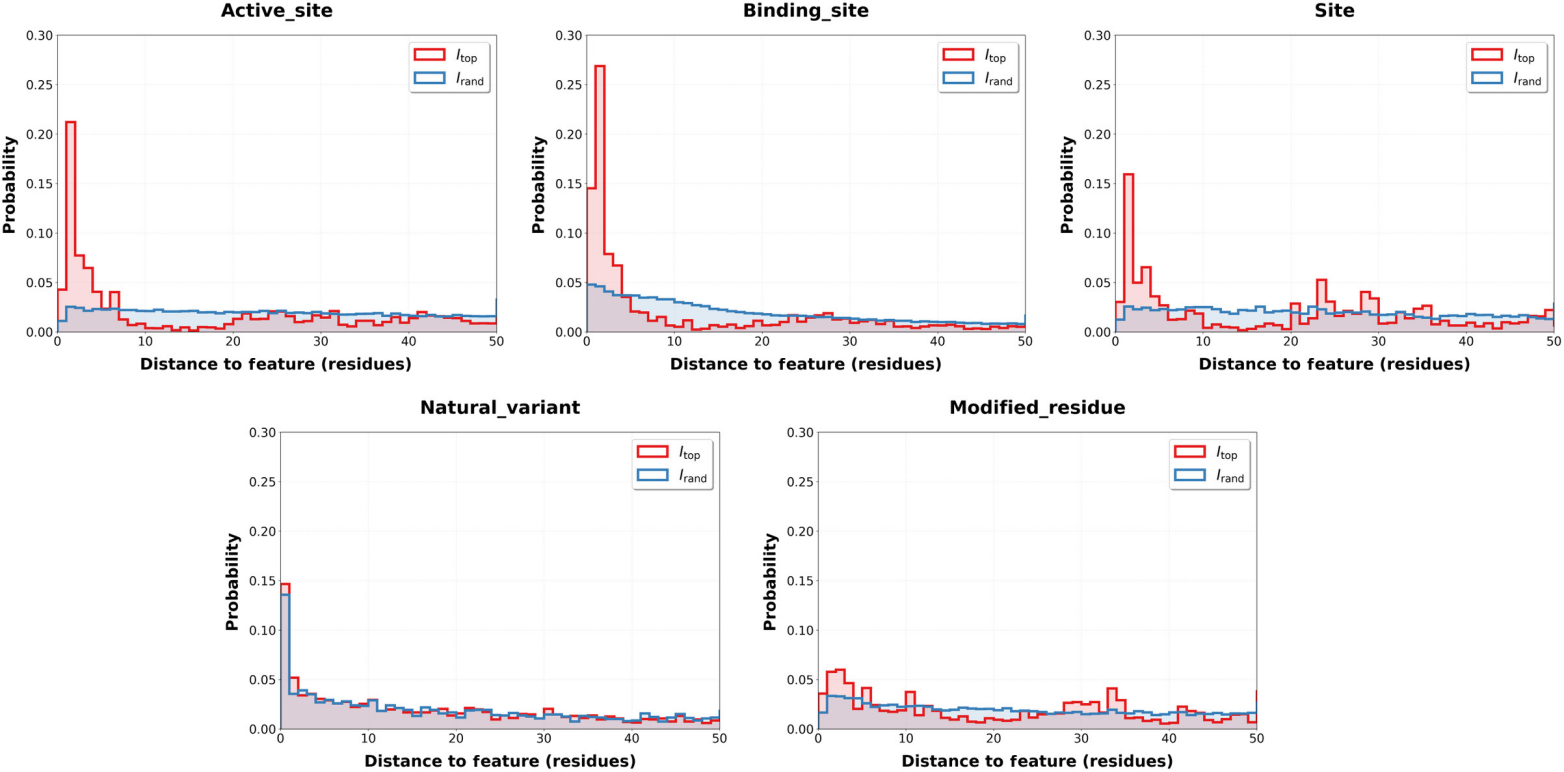
Probability mass functions of the distance from residues to functional sites. The plots show the probability mass functions of the distance from residues with the top 1% attention weights ($I_{\mathrm{top}}$; red) or randomly selected residues ($I_{\mathrm{rand}}$; blue) to residues with specific functional annotations. These functions were calculated across all proteins with functional annotations in the test set.

The heatmaps (Fig. 3A) reveal that the attention weight profiles exhibit distinct peaks along the protein sequences. This suggests a positional proximity between these peaks and the functional sites, although they do not always coincide exactly. Therefore, we next analyzed the sequence distances between the residues with the highest attention weights and their closest functional sites. Specifically, we compared the distance distributions for the residues in the top 1% of attention weights ($I_{\mathrm{top}}$) against those for residues randomly selected from the sequences ($I_{\mathrm{rand}}$) in the test set. For the “Active site”, “Binding site”, and “Site” annotations, the distances between the $I_{\mathrm{top}}$ residues and their closest functional sites were significantly shorter than those calculated for $I_{\mathrm{rand}}$ (Fig. 4). Notably, the probability of a residue annotated as “Active site”, “Binding site”, or “Site” existing within 5 residues of an $I_{\mathrm{top}}$ residue was 45.9%, whereas the corresponding probability for $I_{\mathrm{rand}}$ was only 14.4%. For the “Modified residue” and “Natural variant” annotations, however, the distance distributions for $I_{\mathrm{top}}$ were nearly indistinguishable from those of $I_{\mathrm{rand}}$, indicating that the attention weights show little to no correlation with these specific annotations. Overall, these findings indicate that residues assigned high attention weights tend to be located near annotated functional regions. This observation suggests that the attention mechanism preferentially emphasizes sequence regions that contribute to functional identity prediction.

### Correlation between attention weights and evolutionary conservation

**Fig. 5.**
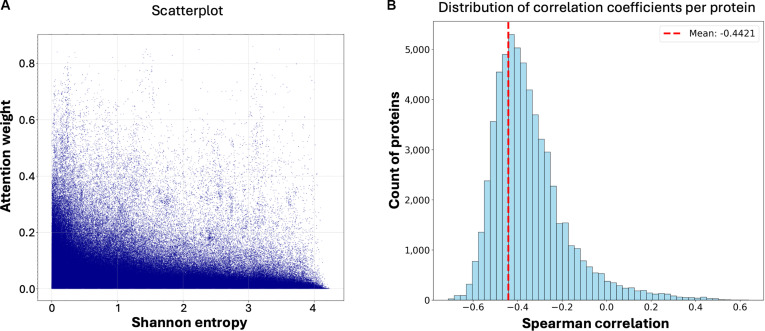
Correlation between attention weights and evolutionary conservation. (A) Scatterplot of per-residue attention weights against Shannon entropies across all proteins in the test dataset. Lower Shannon entropy values indicate greater conservation. (B) Distribution of Spearman’s rank correlation coefficient between Shannon entropies and attention weights calculated for each protein. The dashed red line indicates the mean correlation coefficient of −0.4421.

Since functionally important residues tend to be evolutionarily conserved [30], we assessed the correlation between attention weights and evolutionary conservation using Spearman’s rank correlation coefficients. Evolutionary conservation was quantified using Shannon entropy calculated from the MSA obtained for each protein in the test set. Figure 5A presents a scatterplot of attention weights against entropy for all residues. As discussed in the previous section, higher attention weights reflect functional importance, whereas lower entropy values indicate higher degrees of conservation. Consistently, the plot indicates a negative correlation. The Spearman’s rank correlation coefficients calculated for each protein exhibited a unimodal distribution with a mean value of −0.4421 (Fig. 5B), indicating that attention weights are moderately correlated with evolutionary conservation. Notably, the moderate correlations indicate that the distribution of attention weights cannot be explained solely by evolutionary conservation or annotated functional residues. Because the attention module operates by reweighting residue representations derived from ESM-C embeddings, these results suggest that the model integrates multiple aspects of the pretrained representations when constructing protein-level features, rather than relying exclusively on sequence conservation or known functional sites.

### Performance on low sequence similarity dataset

**Fig. 6.**
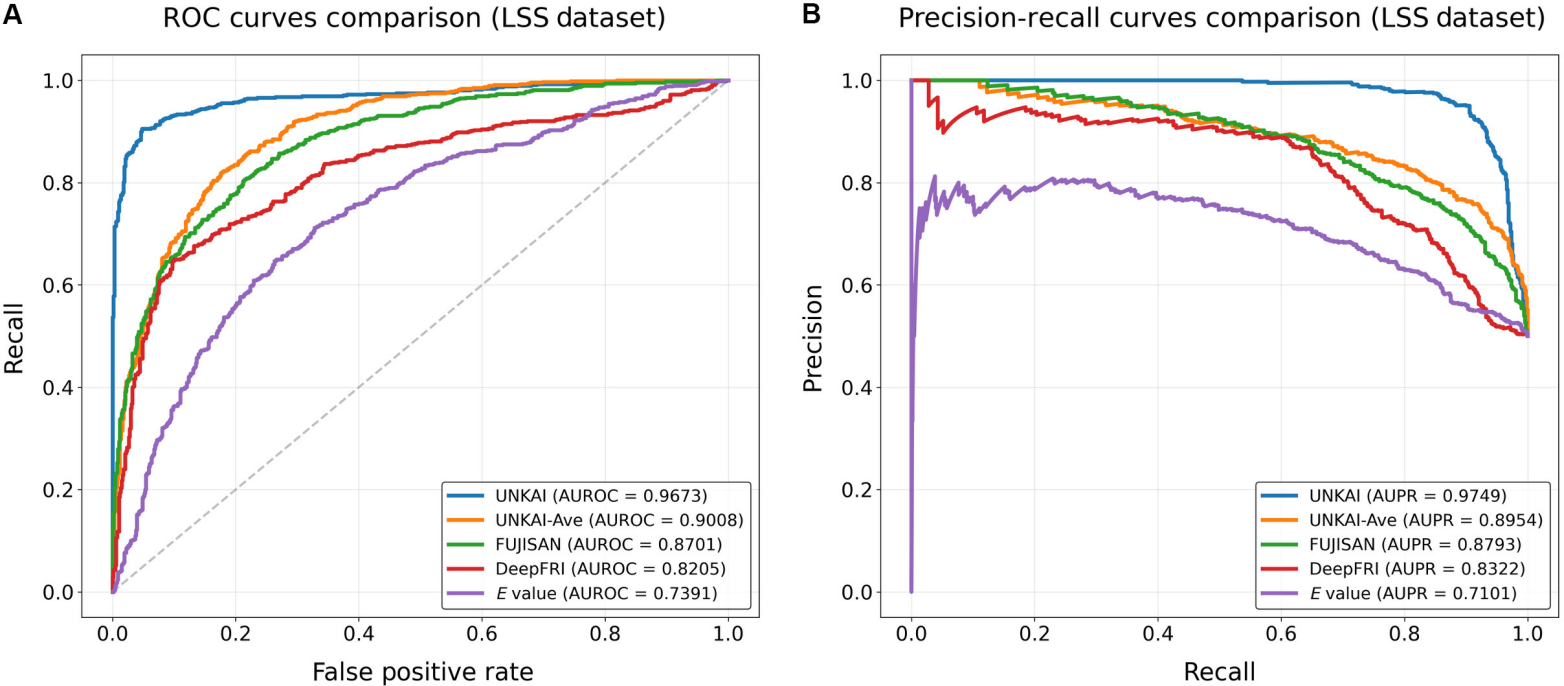
Predictive performance comparison on the low sequence similarity (LSS) dataset. (A) Receiver operating characteristic (ROC) curves and (B) precision–recall (PR) curves for UNKAI and benchmark methods evaluated on the LSS dataset. AUROC and AUPR values for each method are provided in parentheses within the legend. Results of FUJISAN, DeepFRI, and *E* value are cited from Ref. [8]. The dashed diagonal line in (A) represents the performance of a random classifier.

Since UNKAI and FUJISAN were trained on datasets consisting of protein pairs with detectable sequence similarity (*E* value < 10), their predictive performance may inherently depend on the degree of sequence similarity between the input pairs. In our previous study, we demonstrated the robustness of FUJISAN by evaluating its performance on protein pairs with low full-length similarity [8]. To evaluate UNKAI’s performance in this context, we constructed a low sequence similarity (LSS) dataset comprising pairs with *E* values > 10^−10^ (680 pairs with identical functions and 680 pairs with different functions). The LSS dataset was constructed independently from the original training procedure and exhibited only negligible overlap with the original train/validation/test datasets (3 negative protein pairs). Therefore, the evaluation can be regarded as largely independent of the original benchmark. Figure 6A and B presents the ROC and PR curves of UNKAI, UNKAI-Ave, FUJISAN, and other existing methods. Although the AUROC and AUPR values of UNKAI (0.9673 and 0.9749, respectively) were slightly lower than those obtained on the original test set, UNKAI still outperformed all other models on the LSS dataset. These results suggest that UNKAI captures essential functional features that extend beyond simple sequence homology. While alignment-based methods, such as those relying on *E* values, are inherently limited by sequence similarity, our model demonstrates high robustness in identifying functional relationships among protein pairs with low BLAST similarity.

### Performance on sequence identity-based splits

Although the original train/validation/test datasets were split at the protein-pair level, closely related proteins could still be distributed across different subsets, potentially leading to optimistic estimates of prediction performance. To assess the robustness of UNKAI under more stringent conditions, we evaluated the model using sequence identity-based dataset splits. Two evaluation settings were considered. In the strict setting, proteins were clustered according to sequence similarity before dataset construction, and protein pairs were generated exclusively from clusters assigned to the same split. Consequently, no sequence clusters were shared across the training, validation, and test datasets, providing a rigorous assessment of generalization to completely unseen protein families. In the seen–unseen setting, each test pair consisted of one protein originating from a training cluster and another from an unseen cluster, representing a more practical application scenario where only one protein is novel. Under the strict setting, UNKAI achieved an AUROC of 0.9012, an AUPR of 0.8994, and an F1 score of 0.8288 (Table 2). Although these values were lower than those obtained using the original random split, the model retained substantial predictive performance despite being evaluated on completely unseen sequence clusters. In the seen–unseen setting, prediction accuracy was markedly improved, yielding an AUROC of 0.9608, an AUPR of 0.9581, and an F1 score of 0.8976 (Table 2). These results indicate that prediction improves substantially when one protein in a pair belongs to a sequence cluster represented during training. Collectively, these results demonstrate that UNKAI maintains strong predictive performance even under sequence identity-aware evaluation protocols and is capable of generalizing beyond protein families observed during training. The performance difference between the 2 settings further suggests that functional inference becomes considerably more accurate when at least one member of a protein pair belongs to a sequence family represented in the training data.

**Table 2.** Performance on sequence identity-based splits

Dataset	ACC	PRE	REC	F1	AUROC	AUPR
Strict	0.8206	0.7927	0.8682	0.8288	0.9012	0.8994
Seen–unseen	0.8950	0.8758	0.9206	0.8976	0.9608	0.9581

The strict and seen–unseen evaluations should not be regarded solely as benchmark settings but also as practical operating modes. When deploying UNKAI, the sequence relationship between a query protein and the training database can be assessed prior to prediction using sequence clustering or homology searches. This allows the prediction threshold to be dynamically adapted to the expected generalization regime, thereby ensuring more reliable predictions in practical applications. Although performance decreased under the strict evaluation compared with the original random split (Table 1), it remained sufficiently high, demonstrating that UNKAI captures intrinsic functional representations rather than relying on simple memorization of homologous proteins.

### Limitations

In general, prediction accuracy is inherently limited for inputs that lack similar samples in the training set. Although the training set of UNKAI encompasses a wide variety of proteins, the distribution of functional categories—classified at the third digit level of Enzyme Commission (EC) numbers—exhibits some bias, reflecting the distribution of the original database (Fig. S2). To evaluate UNKAI’s predictive capabilities for entirely unseen EC subclasses, we conducted a leave-one-subclass-out evaluation for 5 specific subclasses (Table 3). Notably, predictive performance for these unseen subclasses was lower than the overall performance reported in Table 1. The leave-one-EC-subclass-out evaluation represents an intentionally stringent scenario in which an entire functional category is absent from the training data. Under this setting, the *E* value baseline outperformed UNKAI for most evaluation metrics, indicating that sequence similarity remains a strong indicator of function when homologous proteins are available. Conversely, the reduced performance of UNKAI suggests that the model still has limited ability to extrapolate to completely unseen functional categories, highlighting an important direction for future improvement.

**Table 3.** Predictive performance of UNKAI on unseen EC subclasses

Model ^a^	ACC	PRE	REC	F1	AUROC	AUPR
UNKAI-EC3.6.4	0.7629	0.8301	0.6610	0.7360	0.8489	0.8542
UNKAI-EC2.7.1	0.7388	0.8255	0.6055	0.6986	0.8312	0.8340
UNKAI-EC2.7.4	0.6655	0.6520	0.7102	0.6798	0.7337	0.7302
UNKAI-EC3.6.1	0.7000	0.7560	0.5906	0.6631	0.7614	0.7686
UNKAI-EC4.2.1	0.7553	0.7378	0.7921	0.7640	0.8464	0.8719
*E* value-EC3.6.4	0.8210	0.7379	0.7753	0.8347	0.8929	0.8787
*E* value-EC2.7.1	0.8157	0.7320	0.7704	0.8299	0.8877	0.8768
*E* value-EC2.7.4	0.8190	0.7393	0.7751	0.8323	0.8885	0.8775
*E* value-EC3.6.1	0.8159	0.7333	0.7711	0.8300	0.8883	0.8770
*E* value-EC4.2.1	0.8212	0.7518	0.7820	0.8328	0.8911	0.8804

^a^ The suffix “-ECX.Y.Z” denotes the specific EC subclass that was excluded from the training set and subsequently used for performance evaluation.

Although UNKAI achieved higher performance than the previously reported FUJISAN model, this comparison should be interpreted with caution because the 2 frameworks were evaluated using different datasets and experimental settings. Therefore, the present results do not represent a strict head-to-head benchmark under a unified evaluation protocol. Our objective was not to retrain previously published models under standardized conditions, but rather to compare the performance of methods as they would be available to end users. In this context, the reported results provide a practical indication of the relative performance of publicly available approaches. In contrast, the comparison between UNKAI and UNKAI-Ave was performed using identical training and test datasets. The consistent performance improvement observed for UNKAI suggests that the attention-pooling mechanism contributes substantially to the predictive performance of the model. Nevertheless, direct retraining and evaluation of all methods on standardized datasets would provide a more rigorous benchmark and should be considered in future studies.

The low-sequence-similarity (LSS) dataset was designed to evaluate prediction performance for protein pairs with weak sequence similarity. When constructing the LSS dataset, we minimized overlap with the original datasets at the protein-pair level; however, 3 negative protein pairs remained duplicated. Overlap of individual proteins, homologous proteins, sequence clusters, Rhea annotations, or EC subclasses between the training data and the LSS dataset was not explicitly controlled. Therefore, the current LSS analysis does not fully assess generalization to entirely unseen protein families or functional categories. A more stringent evaluation based on sequence-cluster-disjoint datasets remains an important direction for future work.

The sequence identity-based evaluation introduced provides a more stringent estimate of model generalization than the original random split. Nevertheless, because only UNKAI was evaluated under this protocol, direct comparison with previously published methods under identical conditions remains an important subject for future work.

## Conclusions

In this study, we developed UNKAI, a deep learning-based model designed to predict the functional identity of protein pairs by leveraging ESM-C latent representations of the protein sequences and an attention mechanism. Unlike our previous hypothesis-driven method, FUJISAN, UNKAI adopts a fully data-driven approach to dynamically capture residue-level importance. Our results demonstrate that UNKAI significantly outperforms existing methods, including FUJISAN. This superior performance is largely attributed to the attention mechanism, which computes weighted sums of the ESM-C latent representations of input protein sequences to yield fixed-length aggregated feature vectors. Notably, the attention weights exhibited moderate but significant correlations with both evolutionary conservation and functional annotations—such as “Active site”, “Binding site”, and “Site” from the Swiss-Prot database. These findings indicate that while the attention mechanism effectively captures known functional regions, it also integrates additional information that extends far beyond simple conservation patterns through the learning process. Although challenges remain in generalizing to completely unseen functional classes, this framework provides a powerful tool for the functional annotation of uncharacterized proteins and serves as a foundational step toward more generalized enzyme discovery methods.

## Data Availability

The data and source code supporting this study are available from the corresponding author upon reasonable request.
